# Efficacy of Combined Rifampicin Formulations Delivered by the Pulmonary Route to Treat Tuberculosis in the Guinea Pig Model

**DOI:** 10.3390/pharmaceutics13081309

**Published:** 2021-08-21

**Authors:** Lucila Garcia-Contreras, Vasu Sethuraman, Masha Kazantseva, Anthony Hickey

**Affiliations:** 1 Division of Molecular Pharmaceutics, University of North Carolina at Chapel Hill, Chapel Hill, NC 27599, USA; Vasu-Sethuraman@email.unc.edu (V.S.); Masha-Kazantseva@email.unc.edu (M.K.); ahickey@unc.edu (A.H.); 2 Department of Pharmaceutical Sciences, University of Oklahoma Health Sciences Center, Oklahoma City, OK 73117, USA; 3 Synlogic, Inc., Cambridge, MA 02142, USA; 4 IQVIA, Research Triangle Park, Durham, NC 27703, USA; 5 RTI International, Research Triangle Park, Durham, NC 27709, USA

**Keywords:** rifampicin, tuberculosis, liposomes, nebulization, guinea pigs

## Abstract

Liposomes, as vehicles alone or in combination with rifampicin (RIF) microparticles (RMs), were evaluated as vehicles to enhance the permeation of RIF into granulomas. RIF liposomes (RLs) were extruded through a 0.1 µm polypropylene membrane. RMs were prepared by the solvent evaporation method. Four weeks after infection, guinea pigs (GPs) were assigned to groups treated with a combination of RM-RLs or RLs alone. RLs were nebulized after extrusion whereas RMs were suspended in saline and nebulized to GPs in a nose-only inhalation chamber. Necropsy was performed after the treatment; the lungs and spleen were resected for bacteriology. RLs had mean diameters of 137.1 ± 33.7 nm whereas RMs had a projected area diameter of 2.48 µm. The volume diameter of RMs was 64 ± 1 µm, indicating that RMs were aggregated. The treatment of TB-infected GPs with RLs significantly reduced their lung bacterial burden and wet spleen weight compared with those treated with blank liposomes. The treatment of TB-infected animals with RM-RLs also reduced their lung bacterial burden and wet spleen weight even though these reductions were not statistically different. Based on these results, the permeation of RIF into granulomas appears to be enhanced when encapsulated into liposomes delivered by the pulmonary route.

## 1. Introduction

In its Global Tuberculosis Report 2020, the World Health Organization (WHO) stated that the rate of new tuberculosis (TB) infections is decreasing in the world [1] but the risk of getting infected by *Mycobacterium tuberculosis* (MTB) is more than 20 times higher for patients infected with the human immunodeficiency virus (HIV) [2]. The latest update of the WHO guidelines for the treatment of drug-susceptible TB still recommends the use of the 6-month rifampicin-based regimen (2 months of isoniazid, rifampicin, pyrazinamide and ethambutol followed by 4 months of isoniazid and rifampicin [3]). The unwanted side effects associated with the use of these four antibiotics are widely reported but rifampicin (RIF) appears to have the most severe side effects including hepatotoxicity, enzyme induction and drug–drug interactions [4]. Moreover, the recent success achieved in treating drug-resistant tuberculosis with the Nix-TB regimen of bedaquiline, pretominid and linezolid is also associated with serious toxicity [5]. The main complication in the management of TB in patients co-infected with HIV is the induction of the cytochrome p450 enzymes by RIF because it causes a significant reduction in the body concentration of protease inhibitor drugs, the cornerstone of anti-retroviral therapy [6]. An alternative approach to treat patients co-infected with HIV and TB would be the administration of RIF by the pulmonary route to decrease the drug–drug interaction with protease inhibitor drugs by avoiding enzyme induction. Treating HIV-TB patients with inhaled RIF would enable the use of smaller doses because RIF would be delivered directly to the primary site of infection—the lungs—thus limiting or avoiding systemic side effects.

We previously reported that a single 10 mg dose of aerosolized RIF-loaded poly (lactide-co-glycolide) microspheres (RPLGAs) administered one day before infection significantly reduced the bacterial burden and markers of inflammation in the lungs of TB-infected guinea pigs compared with untreated controls and those receiving a RIF suspension [7,8]. A subsequent study demonstrated that a single dose of aerosolized RPLGAs administered by passive inhalation five days after infection reduced the bacterial burden in the spleen of TB-infected guinea pigs to the same extent as treatment with aerosolized RIF suspensions for 20 consecutive days [9]. However, neither RPLGAs nor a RIF suspension were able to decrease the bacterial burden in the lungs of treated animals. A possible reason for this lack of effect is that *Mycobacterium tuberculosis* (MTB), the causative agent of TB, is harbored inside granulomas in the lungs of TB-infected animals and patients. Granulomas are formed by different types of immune cells at diverse stages of differentiation surrounded by a fibrous cuff and a ring of lymphocytes [10]. This outer layer of the granuloma and its limited blood supply are likely to limit the permeability of drugs inside the granuloma and to reach MTB.

Liposomes have been employed to enhance drug absorption of drugs with poor water solubility including amphotericin B [11], doxorubicin [12] and docetaxel [13]. They have also been considered for the pulmonary delivery of anti-cancer drugs [14,15] and anti-TB drugs [16,17,18,19] as well as for biopharmaceutical compounds such salmon calcitonin [20]. Notably, in September 2018, the FDA approved the use of Arikayce (an amikacin liposome inhalation suspension) for the treatment of lung infections by the *Mycobacterium avium* complex [21]. Liposomes have been employed to enhance drug penetration through the skin [22]. As with the granuloma, the skin has an outer layer that also limits the permeability of drugs: the stratum corneum. Thus, it may be plausible that they could enhance the permeability of RIF into the granuloma. In the present study, we compared the efficacy of RIF-PLGA microparticles with that of RIF liposomes or their combination to decrease the bacterial burden in the lungs of TB-infected guinea pigs.

## 2. Materials and Methods

### 2.1. Preparation and Characterization of RIF Microspheres

RIF microspheres (RMs, 10% RIF loading) and blank microspheres (BMs) were prepared by the emulsion/solvent evaporation method as previously described [9,23,24]. The dispersed phase consisted of RIF (Sigma, St. Louis, MO, USA) and PLGA (75:25, MW = 85,200, glass transition temperature Tg = 50–55 °C, Birmingham Polymers, Birmingham, AL) dissolved in methylene chloride (Mallinckrodt Baker, Paris, KY, USA). The continuous phase consisted of 0.10 *w*/*v* % Pluronic-F68 (Sigma Chemical, St. Louis, MO, USA) dissolved in a mixture of 70% glycerol (Fisher Scientific, Waltham, MA, USA) and 30% phosphate buffer of pH 5.2. The morphology of the resulting microspheres was visualized by scanning electron microscopy (SEM, Model 6300 JEOL, Peabody, MA, USA). The projected area diameter (Dp) of the microparticles and the geometric standard deviation (GSD) were determined by measuring at least 500 particles in a minimum of 10 fields of view from the SEM images using Sigma Scan^®^ software (version 4.0, Jandel Scientific, Corte Madera, CA, USA). The Hatch–Choate conversion was used to calculate the mass median diameter (MMD) from the Dp [25]. The volume diameter (Dv) of the powders formed by the RMs was obtained by laser diffraction (HELOS particle size analysis, H0838, Sympatec, GMBH, Clausthal-Zellerfeld, Germany). The RM powders were placed on a vibrating feeder operated at a feed rate of 35% of the maximum rate following a shear dispersion of 3.0 bar (RODOS, Sympatec, GMBH, Clausthal-Zellerfeld, Germany).

### 2.2. Liposome Preparation and Characterization

In a round-bottomed flask, soy phosphatidylcholine (L-α-phosphatidylcholine) and cholesterol (1:1 molar ratio, Avanti Polar Lipids, Alabaster, AL, USA) were dissolved in 2.5 mL of chloroform with RIF for an initial concentration of 25 mg/mL. The solvent was then evaporated employing a rotary evaporator (Rotovap RE121, Buchi, Flawil, Switzerland) for 3 h at 125 rpm and 20 °C. The flask containing the lipid film was put into vacuum desiccators in the presence of dry silica gel for 2 h to ensure the complete evaporation of the chloroform. The thin lipid layer was then hydrated with 6 mL of phosphate buffer (pH = 7.4) and annealed for 1 h at 45 °C. To produce unilamellar vesicles [26], RIF liposomes were then extruded (Thermobarrel Extruder, Lipex Biomembranes, Inc., Vancouver, British Columbia, Canada) 10 times through two (0.1 µm, stacked) polycarbonate filters (Osmonics Inc., Livermore, CA, USA) at 170 psig with nitrogen gas. The final RIF concentration of the liposomal suspension was 10 mg/mL.

The vesicle size and distribution were analyzed in an aqueous dispersion by dynamic light scattering (DLS) using a laser particle sizer (Model 370, Nicomp Particle Sizing Systems, Santa Barbara, CA, USA). The liposome samples (10 µL) were diluted with 990 µL PBS for the analysis. As RIF is poorly soluble in water, it was assumed that it would be readily inserted into the liposomal bilayer; thus, the RIF encapsulation efficiency was not calculated. The liposomal formulation was freshly prepared and used directly to dose the animals.

### 2.3. Selection of a Nebulizer and the Characterization of the Nebulizer Output

The size and distribution of the aerosol droplets generated by two nebulizers, the Acorn II nebulizer (Marquest Medical Products, Inc. Englewood, CO, USA) and the Pari LC Star (PARI Respiratory Equipment, Inc., Midlothian, VA, USA), were determined by inertial impaction using 0.01% sodium fluorescein solutions. The aerosols were sampled at 28.3 L/min by an Andersen 1ACFM NonViable Ambient Particle Sizing Sampler (Thermo Electron, Waltham, MA, USA). The T-piece of the nebulizer was connected to a USP throat of the cascade impactor [27] and the solutions were nebulized at 40 psig for 10 min. All runs were performed in quintuplicate (*n* = 5).

### 2.4. Animals

All animal procedures were approved by the University of North Carolina Chapel Hill Institutional Animal Care and Use Committee (IACUC #: 98-130.0, approved on May 1998). Specific pathogen-free male Dunkin Hartley guinea pigs (GPs, Hilltop, Scottsdale, PA, USA) weighing 417 ± 51.6 g were housed individually in a biosafety level 3 (BL-3) containment area with a 12 h light/dark cycle. The animals were allowed free access to water and food (Prolab guinea pig 5P18, PMI feeds, Inc., St. Louis, MO, USA) at all times.

### 2.5. Respiratory Infection

GPs were infected via the respiratory route with a small inoculum (2 × 10^5^ CFU/mL) of *M. tuberculosis* strain H37Rv [8,9]. GPs were placed randomly in an exposure chamber and aerosols were generated by pumping compressed air through a modified MRE-type 3 jet Collison nebulizer (Waltham, MA, USA) containing 5 mL of bacterial suspension.

### 2.6. Treatments

Four weeks after infection, the animals were randomly assigned to five treatment groups (Table 1): a combination of RIF microspheres and RIF liposomes (RM-RLs), RIF liposomes (RLs), a combination of blank microspheres and blank liposomes (BM-BLs), blank liposomes (BLs) and untreated controls. The animals were treated for 10 days as described in Table 1. RIF liposomes were prepared freshly every day of the treatment and aerosolized after a particle size measurement. Microspheres (RMs or BMs) were suspended in 5 mL 0.05% tween 80 saline solution before aerosolization. To receive the treatment, the animals were placed in the ports of a nose-only inhalation chamber (ADG Developments, Ltd., Herts, UK) and formulations (RMs, BMs, RLs or BLs) were aerosolized with an Acorn II nebulizer at 40 psig for 10 min, 5 times (50 min of treatment) each day.

### 2.7. Necropsy Procedure and the Assessment of the Number of Viable Bacteria

GPs were euthanized by an i.p. lethal dose of sodium pentobarbital 39 days after infection (after 10 days of treatment). The lung and spleen wet weights of the TB-infected animals after the respective treatments were used as an indicator of the degree of inflammation of each organ. The larger the organ weight, the greater the extent of the inflammation due to cell infiltration; the smaller the organ weight, the least tissue inflammation, which was considered to be a beneficial effect of the treatment.

After each animal was euthanized and no further breathing was observed, 10–13 mL of blood was withdrawn from the animal by a cardiac puncture before the chest cavity was exposed to avoid the presence of blood in the chest cavity when the lungs were excised. Once resected, the lungs were placed in a petri dish, rinsed with sterile saline and patted dry with a gauze before being weighed and inspected to determine the degree of inflammation and primary lesions. The peritoneal cavity was then opened and the spleen resected and inspected for any remaining connective tissue and primary lesions before being rinsed, patted dry and weighed.

The lower left lobe of the lung, a portion of the spleen and a portion of the liver were resected using separate sterile instruments and stored in 10% neutral buffered formalin for the histopathological analysis. The lower right lobe and a portion of the spleen were homogenized separately in a sterile saline solution. After proper dilutions, aliquots of 0.1 mL were inoculated in M7H10 agar plates. The plates were incubated at 37 °C for 21 days and the number of viable bacteria were counted (colony forming units, CFU).

### 2.8. Statistical Analysis

Data for the particle size analysis and inertial impaction were analyzed by an ANOVA (SAS/STAT, Cary, NC, USA) and the difference between groups (*p* < 0.05) was determined by the least square difference test. Data for the wet organ weight and bacterial burden were analyzed using Graph Pad Prism 8.0 software. A comparison of each treatment group (RM-RLs versus RLs) with their respective blanks (BMs and BLs) and the untreated control group was performed using a non-parametric ANOVA (Kruskal–Wallis test) with a Dunnett’s multiple comparison test to determine which treatments were significantly different from each other. The *p*-values for the primary analysis and the adjusted *p*-values for the multiple comparisons < 0.05 were considered statistically significant.

## 3. Results

### 3.1. Preparation and Characterization of the RIF Formulations

**Figure 1:**
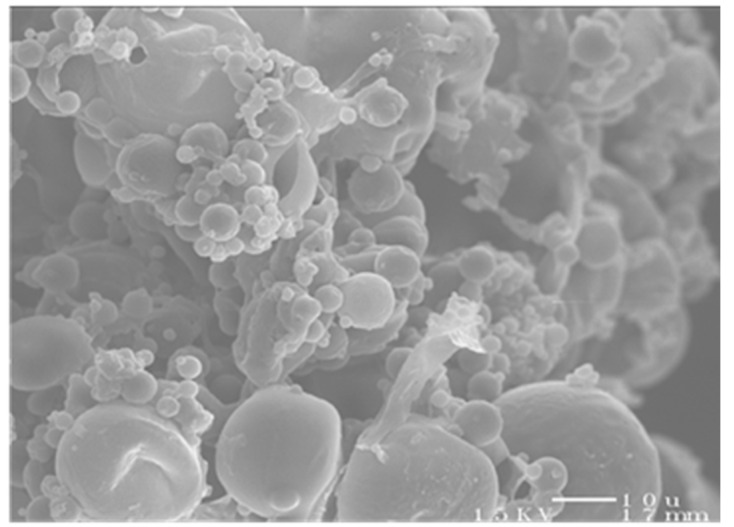
Rifampicin-loaded microspheres prepared by solvent evaporation with Pluronic-F68 as the emulsifier (Note that the bar on the right bottom corner of the image is equivalent to 10 μm, which supports the size of microspheres measured by laser diffraction).

The morphology of the RMs and BMs was similar, having a spherical shape and smooth surface, but several RMs exhibited bridging between individual particles (Figure 1). An image analysis determined that RMs had a Dp = 2.48 µm and the GSD = 1.89 whereas BMs had a Dp = 4.34 µm and the GSD = 1.51. The MMD of the RMs was 8.36 µm and the BMs was 7.22 using the Hatch–Choate conversion. However, the size analysis by DLS indicated that the Dv of the RM powder was 64 ± 1 and the GSD = 2.4 ± 0.9 whereas the Dv of the BM powder was 62 ± 1 and the GSD = 1.8 ± 0.1. Thus, the statistical difference between the Dp and Dv (*p* < 0.05) indicated that the powder was aggregated with strong inter-particulate forces because the shear force provided by the RODOS was not sufficient to disperse the aggregates into primary particles.

After the extrusion, the resulting unilamellar populations of suspension-based liposomes had mean diameters of 137.1 ± 33.7 nm (average of the liposomes prepared for the 10 days of treatment). After sizing, the liposomes were administered to the animals as prepared. The final concentration of RIF in the liposomal suspension was 10 mg/mL. Table 2 summarizes the characteristics of the RIF microspheres and liposomes employed in the study.

### 3.2. Selection of a Nebulizer and the Characterization of the Nebulizer Output

An analysis of the cascade impaction data obtained from the aerosol generated by the studied nebulizers determined that the aerosol droplets produced by the Acorn II nebulizer and the PARI nebulizer were significantly different in size and distribution (*p* < 0.05). The aerosol droplets produced by the Acorn II nebulizer had a mean mass aerodynamic diameter (MMAD) of 1.5 ± 0.06 µm and a GSD = 3.0 ± 0.24 whereas the PARI nebulizer produced droplets with an MMAD = 1.2 ± 0.04 µm and a GSD = 2.3 ± 0.08. Therefore, the Acorn II nebulizer was selected to administer the RMs as aerosols to the animals in the study because it produced a slightly larger droplet size and had a broader size distribution, which could better accommodate the size and distribution of the RMs.

### 3.3. Wet Organ Weights

**Figure 2:**
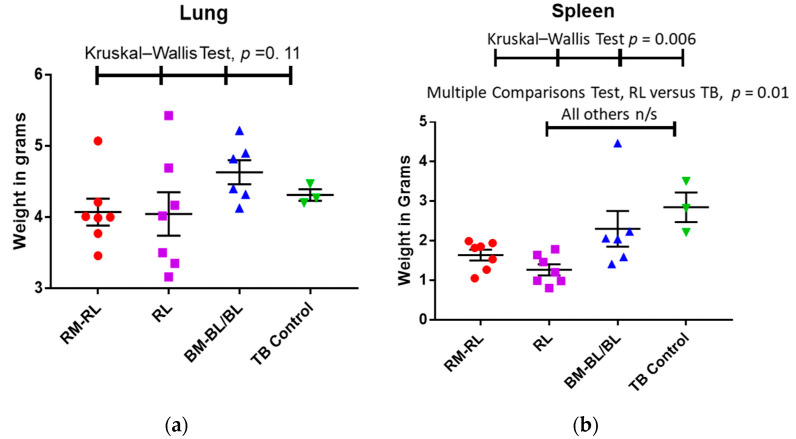
Wet organ weights of TB-infected animals as a function of the treatment (Each colored symbol represents the individual weight of the organ of one animal, whereas the black lines indicate mean ± standard deviation, *n* = 3–7). The tests employed to determine the statistical differences and their corresponding *p*-values are shown at the top of each graph. (**a**) Lung wet weight in grams per body weight; (**b**) spleen wet weight in grams per body weight.

Under the assumption that the larger the organ weight, the greater the extent of inflammation, a smaller organ weight would be considered as a beneficial effect of the treatment. Under this assumption, the average effect of the treatment with RMs and RLs or with RLs alone on the lung tissue weight were similar (not significantly different, *p* = 0.11) when compared with the blanks or the untreated controls (Figure 2a). The wet lung weights of the animals receiving the BM-BLs were similar to those of the animals receiving the BL treatment (*p* = 0.096) and, thus, they were grouped together. A large variability was observed in the weights of the treated groups (RM-RLs and RLs) with the most variable weights observed in the group of the animals treated with RLs, which included the smallest wet lung weight (3.5 g) and the largest wet weight (5.43 g). These differences in wet lung weight remained even after the wet lung weight was corrected by the animal’s body weight (0.82 and 1.54, respectively).

In contrast, the range of wet weights of the spleens of the treated animals in the study was very narrow with the mean spleen weight of the animals treated with RLs being one half (1.3 ± 0.6 g) of that of untreated controls (2.85 ± 1.2 g). The wet spleen weights of the animals receiving the BM-BLs were similar to those of the animals receiving the BL treatment (*p* = 0.248) and, thus, they were grouped together. The homogeneity and difference between these values allowed a declaration of a significant difference (*p* = 0.01) between the animals treated with RLs and the untreated controls by the multiple comparison test (Figure 2b).

### 3.4. Bacteriology of the Lung and Spleen of Guinea Pigs

**Figure 3:**
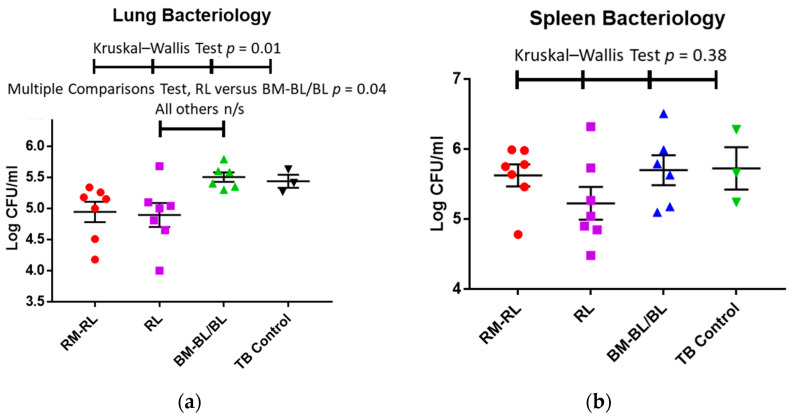
Number of viable bacteria (CFU/mL) in the lung (**a**,**b**) spleen tissues of the animals at necropsy after 10 days of treatment (Each colored symbol represents the individual weight of the organ of one animal, whereas the black lines indicate mean ± standard deviation, *n* = 3–7). The tests employed to determine the statistical differences and their corresponding *p*-values are shown at the top of each graph.

Figure 3 depicts the bacterial burden (log CFU/mL, number of viable bacteria) in the lungs and spleens of the animals in the different treatment groups at the time of necropsy. As with the wet organ weights, there was a large variability in the bacterial burden of the animals treated with the RM-RL combination and those treated with RLs alone. The bacterial burden in the lungs of the animals receiving the BM-BLs were similar to those of the animals receiving the BL treatment (*p* = 0.912) and, thus, they were grouped together. Compared with the blanks and untreated controls (Figure 3a), the bacterial burden in the lungs of the animals treated with the RM-RL combination and RLs alone were 0.6–0.7 CFU/mL smaller but due to the variability in the RM-RL group, only the bacterial burden of the RLs-alone treated group was significantly smaller (*p* = 0.04) than that of the group treated with blanks (BM-BLs) by the multiple comparison test.

The bacterial burden in the spleens of the animals receiving the BM-BLs was similar to those of the animals receiving the BL treatment (*p* = 0.186) and, thus, they were grouped together. The mean bacterial burden in the spleen of the animals treated with RLs alone was smaller (5.2 CFU/mL) than that of any other group in the study (5.6–5.7 CFU/mL) but the large variability of the bacterial burden among individual animals of each group (Figure 3b) influenced the statistical analysis of these results.

## 4. Discussion

The widespread use of newer and more effective anti-retroviral therapies has decreased the mortality of HIV patients since AIDS was first reported in the early 1980s. However, a retrospective study based on the autopsies of HIV-infected patients who died at a major New York City hospital from 1984 to 2016 revealed that 86% of deaths from 2012–2016 were due to opportunistic infections including pneumonia and TB [28]. The fragile health state of HIV patients may also make them more susceptible to the side effects of conventional TB treatments, particularly those related to the oral use of RIF such as nausea, vomiting, a rash, fever or difficulty swallowing or breathing. Thus, alternative routes of administration for RIF are highly desirable.

Previous publications reported that RMs prepared by our group could provide a sustained release of RIF in the lung environment [23] and were effectively phagocytosed by alveolar macrophages [29]. Subsequent studies showed that these RMs reduced the bacterial burden in the lungs of animals when administered prior to infection [7] or reduced the bacterial burden in the spleen of animals when administered 5 days before infection [9]. However, RMs had no effect on the bacterial burden of animals in which the TB infection was fully established (4 weeks after infection) [9].

Liposomes are formed using lipids such as cholesterol and dipalmitoyl-phosphatidyl-choline (DPPC), which is present in the lung surfactant [20]. They are often considered to be vehicles that encapsulate compounds that are associated with systemic toxicity in order to improve their therapeutic index [15] and enhance drug penetration through the skin [22]. We hypothesized that a combined treatment with RMs and RLs could provide alveolar macrophage targeting, a sustained release of RIF and enhance its permeation into the granuloma. The present study was undertaken to evaluate the use of liposomes as vehicles alone or in combination with RMs to enhance the permeation of RIF through the fibrous cuff that surrounds the granuloma and improve the efficacy of a TB treatment with inhaled RIF.

The results of the lung bacteriology of the present study appear to suggest that liposomes may have enhanced RIF permeation in the granulomas that were present in the lungs of guinea pigs with an established TB infection (4 weeks). The treatment of these animals with RLs for 10 days significantly reduced their lung bacterial burden and wet spleen weight compared with those treated with blank liposomes. The treatment of the TB-infected animals with RM-RLs also reduced their lung bacterial burden and wet spleen weight even though these reductions were not statistically different.

Several publications describe the encapsulation of anti-TB drugs into liposomes such as isoniazid [30,31], RIF [32,33,34,35], pyrazinamide [36], rifabutin [37], clofazimine [38] and combinations of several of these drugs [16,36,39,40]. The purpose of these liposomal formulations includes the targeting of macrophages to achieve a sustained release of the drug or decrease the systemic toxicity of the specific drug. Most of these studies focus on the development and optimization of the formulation in terms of the lipid composition, methods of preparation to maximize the encapsulation efficiency and their stability [16,30,31,32,33,34,35,36,37,38,39,40]. In addition to the formulation, a handful of these studies have determined the biodistribution of these formulations in laboratory animal models [16,35,36] and a few of these studies evaluate their efficacy in animal models of TB [31,37,38,39,40,41,42]. However, only one of these studies evaluated the liposomal formulations in guinea pigs [40] and only one administered the formulation by the pulmonary route [42].

Most of the liposomal formulations described above consisted of multilamellar vesicles composed of 2–4 lipids. A few of these formulations included PEG to make the liposomes stealthy [31,39,40]; others added compounds such as mannan [36] and O-steroyl amylopectin [31,35,39,40] for lung targeting or maleylated bovine serum albumin for alveolar macrophage targeting [35]. In contrast, the formulation employed in the present study consisted only of soy phosphatidylcholine (a compound similar to the lipid present in the lung surfactant) and cholesterol (to modify and stabilize the fluidity of the liposomal bilayer [34,43]). This formulation was similar to Lipoquin^®^(Aradigm Corp., Hayward, CA 94545, USA), which was reported to provide sustained levels of ciprofloxacin in the lungs for 24 h [44].

The route of administration and the mode of administration are also important for the efficacy of the treatment with liposomal formulations. The majority of the published studies described above administered the liposomal formulation by IV injection [31,37,38,39,40]. While these studies reported an efficacy in the TB animal model, several of these studies reported significant drug liposome concentrations in the liver and spleen and smaller drug concentrations in the lungs of treated animals [31,37]. In addition, the uptake of liposomes by the reticuloendothelial system was reported to be up to 52% of the original dose [31]. In contrast, the RLs in the present study were delivered directly to the lungs of infected animals by nebulization thus ensuring that all the RLs were delivered to the lungs, the main site of the TB infection.

Nebulization can be detrimental to liposome formulations because the shear forces and the formation of widespread air–liquid interfacial surfaces can disrupt the integrity of multilamellar vesicles and large unilamellar vesicles and release the entrapped drug [45]. The extent of the liposomal disruption is influenced by the lipid composition [46], the size of the liposome [47] and the operating conditions of the nebulizer [48]. The RL formulation employed in the present study contained soy phosphatidylcholine, which has a high phase transition temperature, and cholesterol, which is known to provide stability to liposomes [46]. Moreover, the structure (unilamellar) and size of the RL formulation (~137 nm) have been reported to be minimally susceptible to disruption by nebulization [44].

In the present study, the extent of the reduction of the bacterial burden in the lungs of the guinea pigs treated with RLs was modest (0.7 CFU/mL) compared with the reduction of the bacterial burden of mice treated by the IV route with liposomal formulations of rifabutin (1.0 CFU, [37]), isoniazid (1.3 CFU, [31]), rifampicin (1.2 CFU, [31]) and the combination of isoniazid and RIF (1.0 CFU, [39]). Likewise, the reduction of the bacterial burden in the spleen of guinea pigs in the present study was also modest (0.4–0.5 CFU) compared with that of mice treated by the IV route with liposomal formulations of rifabutin (2.1 CFU, [37]), isoniazid (0.6 CFU, [31]), rifampicin (0.7 CFU, [31]) and the combination of isoniazid and RIF (1.0 CFU, [39]). Pandey et al. [40] reported a 1.7 CFU reduction in the “organs” of guinea pigs treated with isoniazid-RIF liposomes by the IV route but the organs that the authors refer to were not specified. The differences in the extent of the reduction of the bacterial burden between the present study and previously published studies can be explained by the differences in the animal model, the route of infection, the route of the treatment administration, the length of the treatment and the dose of the drug administered or delivered. Guinea pigs are known to be exquisitely susceptible to MTB infection by inhalation [49] whereas mice are more resistant to infection and several bacteria injected by the IV route may be cleared systemically before colonizing their lungs. In the present study, the animals were treated daily for 10 days starting 4 weeks post-infection whereas the mice in the published studies were treated at an earlier stage of infection and for a longer period of time. For example, mice were treated with rifabutin for 14 days starting 3 weeks post-infection [37], mice treated with isoniazid alone and RIF alone were treated twice a week for 6 weeks starting 2 weeks post-infection [31] and mice treated with the isoniazid-RIF combination were treated once a week for 6 weeks starting 2 weeks post-infection [39]. Guinea pigs in the study by Pandey et al. [40] were infected by the IM route and it is not clear if the bacteria were able to colonize the lungs or spleen of these animals or to what extent. These guinea pigs were treated i.v. with the liposomal drugs weekly (7 doses) for 6 weeks starting 20 days after infection [40]. It is important to note that during IV treatments to the mice, the whole drug dose (10–20 mg/kg of body weight) was administered into the systemic circulation whereas the dose administered to the guinea pigs in the present study was a fraction of that administered to the mice. Considering the volume and concentration of the RL suspension, the rate at which the nebulizer was operated, the respirable fraction of the aerosol (for guinea pigs) as well as the tidal volume and breathing frequency of the animal and the length of the treatment, the animals in the present study inhaled a dose of 2.83 mg/kg. This inhaled dose is 3–8 times smaller than the IV doses employed in the published mice studies.

Lastly, animals treated with the RM-RL combination had a similar or smaller decrease in wet tissue weights and the bacterial burden in their lungs and spleen compared with those receiving the RL-alone treatment. This was likely due to the large fraction of the RMs (~0.85) that were bigger (due to the aggregation of individual microparticles) than the droplets of the buffer produced by the Acorn nebulizer. Thus, the liquid in the nebulizer was aerosolized but only a fraction of the aerosol droplets delivered to the animals contained RMs. To avoid these limitations of the treatment with RMs, future studies should employ microparticles manufactured by spray drying that would have smaller sizes and less aggregation or they should be delivered as dry powders. Several research groups have developed dry powder formulations of liposomes [17,18,32,33], which may enhance the efficiency of the aerosol delivery of anti-TB drugs.

## 5. Conclusions

The results of the present study suggest that the permeation of RIF into granulomas may be enhanced when delivered as a liposomal formulation by the pulmonary route, as suggested by a modest decrease in wet organ weights and the bacterial burden in the lungs and spleen of animals with an established TB infection. Additional studies that would determine the drug concentrations in the granuloma versus the unaffected lung tissue in the same animal would provide definite evidence to support our conclusion. Future studies should consider longer treatment times (>10 days) and the use of additional anti-TB drugs, preferably those acting on the cell wall of the bacteria such as isoniazid or pyrazinamide, to fully assess the efficacy of this approach to treat TB. It may be possible that the use liposomal formulations can be extended to other water insoluble anti-TB drugs to enhance their permeation into granulomas or drugs that have severe side effects. Such liposomal formulations could be also used in combination with other optimized formulations (inhalable dry powders or existing oral therapies) to improve the efficacy of existing treatments.

## Figures and Tables

**Table 1:** Summary of the treatment groups with either rifampicin liposomes (RLs) or a combination of rifampicin liposomes and rifampicin microspheres (RMs). Negative controls consisted of groups treated with either blank liposomes (BLs) or a combination of blank liposomes and blank microspheres (BMs) or untreated controls.

Treatment Day	Treatment Groups				
	RIF Formulation (RM ^1^ Or RL ^2^)		Negative Controls		
1	RM	RL every day	BM	BL every day	Untreated controls
2	RM		BM		
3	RM		BM		
4	RL		BL		
5	RM		BM		
6	RL		BL		
7	RM		BM		
8	RL		BL		
9	RL		BL		
10	RL		BL		

^1^ RMs consisted of 10% RIF; thus, when treated with RMs, the animals received 20 mg of RIF per day. ^2^ RLs consisted of 10 mg/mL RIF; thus, when treated with RLs, the animals received 250 mg of RIF per day.

**Table 2:** Characteristics of the RIF formulations employed to dose TB-infected guinea pigs.

Property	RIF Liposomes	RIF Microspheres
Size (µm)	0.1371 ± 0.0337	Dp = 2.48; Dv = 64
Polydispersity or GSD	n.d.	1.89; 2.4
Drug loading	20%	10%

## Data Availability

Not applicable.
